# The effect of various therapeutic exercises on forward head posture, rounded shoulder, and hyperkyphosis among people with upper crossed syndrome: a systematic review and meta-analysis

**DOI:** 10.1186/s12891-024-07224-4

**Published:** 2024-02-01

**Authors:** Simin Sepehri, Rahman Sheikhhoseini, Hashem Piri, Parisa Sayyadi

**Affiliations:** 1 Faculty of Physical Education and Sport Sciences, Allameh Tabataba’i University, Tehran, Iran; 2 Department of Corrective Exercise & Sport injury, Faculty of Physical Education and Sport Sciences, Allameh Tabataba’i University, Western Azadi Sport Complex Boulevard, Hakim Highway, Tehran, Iran; 3 Department of Health and Sport Medicine, Faculty of Physical Education and Sport Sciences, University of Tehran, Tehran, Iran

**Keywords:** Upper crossed syndrome, Forward head, Forward shoulder, Round shoulder, Kyphosis

## Abstract

**Objectives:**

This review study aimed to evaluate the impact of therapeutic exercises on Upper-Crossed Syndrome (UCS). The study utilized a systematic review and meta-analysis approach to investigate the effects of various therapeutic exercises on forward head posture, rounded shoulders, and hyperkyphosis associated with upper crossed syndrome.

**Methods:**

The study identified relevant keywords for each independent and dependent variable and conducted a search in scientific databases, including PubMed, Web of Science, Scopus, and Google Scholar, without any time limitations until 12 August 2023. Overall, 4625 articles were found in the selected databases, which were reduced to 1085 after being entered into the EndNote software and removing duplicate data. The full texts of 30 remaining studies were reviewed; ten articles meeting the criteria were included. Additionally, 12 studies from the Google Scholar database were included, resulting in 22 studies. Using Comprehensive meta-analysis software (CMA ver 3), data heterogeneity was measured with I^2^ and the Q tests. The Funnel Plot and Egger test methods were utilized to determine the possibility of publication bias. The JBI checklist was used to assess the quality of the studies.

**Results:**

The results of the meta-analysis showed that therapeutic exercises were effective in improving forward head, rounded shoulders, and thoracic kyphosis angles (CI 95% = -1.85–1.161, *P* = 0.001, *P* = 0.001, CI95%=-1.822–1.15, and *P* = 0.001, CI 95%= -1.83–1.09, respectively).

**Conclusion:**

Based on the results, it appears that performing therapeutic exercises in the form of strength exercises, stretching, shoulder-based exercises, and incredibly comprehensive exercises that target all muscles may be effective in reducing forward head, rounded shoulders, thoracic kyphosis, and overall UCS.

**Level of evidence:**

1

**Supplementary Information:**

The online version contains supplementary material available at 10.1186/s12891-024-07224-4.

## Introduction

Upper Crossed Syndrome (UCS) is characterized by upper quarter abnormalities, such as increased thoracic kyphosis, rounded shoulders, and forward head posture [1, 2]. The prevalence of UCS ranges from 11 to 60% in different populations and age groups [3]. This disorder affects the upper quarter of the body and has multiple causes [2, 4]. Musculoskeletal abnormalities may develop over extended periods due to biomechanical, psychological, and social stresses and repetitive activities [5, 6]. Repetitive movements can cause muscle length, strength, and stiffness changes, which may lead to movement disorders [7]. Some adverse effects of UCS include early fatigue, pain in the back, neck, and shoulder segments, decreased respiratory capacity and increased residual volume, reduced aerobic endurance, an unattractive appearance, and vertebral fractures [8]. This highlights the importance of preventing and correcting this postural abnormality [7].

In addition, it has been observed that UCS can lead to a chain reaction of disorders in more distant segments, such as the lower limbs [1, 2]. For instance, an increase in lumbar hyper-lordosis resulting from changes in the thoracic hyper-kyphosis and cervical hyper-lordosis is one of the indicators of this chain reaction phenomenon, which affects the back muscles and anterior thigh muscles [9]. Therefore, selecting appropriate exercises to correct UCS and prevent further disorders is crucial. In this regard, several studies have explored the effectiveness of various therapeutic exercises for this condition, including therapeutic exercises in games, strength training, stretching exercises, shoulder-specific exercises, and comprehensive exercises [10–13].

UCS is characterized by tightness in specific neck muscles and weakness in others [14]. Therapists emphasize the importance of assessing the head, shoulders, and spine position because misalignment in these areas can affect several biomechanical variables, motor control, and performance [15]. Exercise is assumed to correct existing muscular disorders [16]. However, despite postural correction interventions being widely included in exercise interventions [16–19], there is limited or conflicting empirical data to prove the effectiveness of exercise. Additionally, little information about which exercise is the most effective intervention [1, 20]. In this line, two systematic and meta-analytical reviews have shown that therapeutic exercise can improve inappropriate postural control in individuals with other postural misalignments [19, 21]. Therefore, exercise practitioners require evidence-based recommendations for effective exercises for individuals with postural deviations. Moreover, it is essential to be aware of practical and effective intervention strategies with detailed descriptions to improve inappropriate postural control or prevent its consequences.

The UCS is one of the prevalent postural malalignments in society, which various physical disorders and symptoms may accompany. Therefore, therapeutic exercise has been suggested to improve posture in UCS individuals. Despite several studies investigating the efficacy of therapeutic exercises for UCS, discrepancies persist among their findings. Consequently, prescribing therapeutic exercise programs to enhance the posture of people with UCS requires more robust scientific evidence. Thus, a systematic review and meta-analysis are necessary to provide a comprehensive summary and make definitive conclusions. Consequently, this systematic review aimed to compile and assess the effectiveness of various therapeutic exercises on forward head posture, rounded shoulder, and hyperkyphosis among people with upper crossed syndrome.

## Methods

### Search strategy

The study at hand follows the Cochrane guidelines and PRISMA checklist, and it is a systematic review and meta-analysis. A search strategy was employed to identify eligible articles, searching for English and Persian articles using specific keywords. The keywords were grouped with AND between each group, and OR was used between the keywords within each group. The search terms used for the search were: (“Upper crossed syndrome” OR “Upper-crossed syndrome” OR “Forward head” OR “rounded shoulder” OR hyperkyphosis OR “thoracic kyphosis”) AND (Exercise OR Training OR Protocol OR Rehabilitation OR “physical therapy” OR “therapeutic exercise” OR “exercise therapy” OR “Exercise Movement Techniques” OR physiotherapy). The searches were performed in the SCOPUS, PUBMED, and Web of Science databases. Moreover, the eligible paper citations and Google Scholar were searched as additional sources to find the relevant sources. To conduct a search in the Google Scholar database, only the terms “Upper Crossed Syndrome,” “Exercise,” and “Training” were queried using the AND operator, and the identified sources were subjected to examination. The articles considered for this study were original, full-text articles published in peer-reviewed journals, and they must meet the following criteria: written in either Persian or English, with no restrictions on publication date, focused on the efficacy of a common exercise program for individuals with UCS, investigated at least one of the dependent variables of interest in the present study, and had an intervention program duration of at least three weeks. There was no time limitation for the search strategy and the searches were done on 6 July 2023.

### Search process

Initially, a search was conducted in selected databases, and the information, including author names, titles, and abstracts, was entered into an EndNote file. After removing duplicate records, two researchers independently reviewed the studies while retaining the original author names, titles, and abstracts. Any discrepancies were resolved by the group supervisor, who acted as the final reviewer. The full-text articles that met the inclusion criteria for this study, including the name of the first author, publication year, quality, sample size, participants’ characteristics (age, gender, relevant indices, etc.), intervention and exercise specifications (type, intensity, duration, etc.), methods and tools for data collection, and the most important results obtained, were summarized in Table 1.

**Table 1:** Characteristics of included studies, NA; not available

Author and Year	Participants	Number of participants (Male/Female) (Age ± Standard Deviation)	Interventions	Comparison	Duration of Exercise (weeks), (day/week), (Time/session (min))	Variable / Condition	Result
Salamat et al., 2020 [22]	Students	Corrective Functional Exercises: 12 (12/0) (11.5 ± 1.16) Corrective Plays: 12 (12/0) (11.33 ± 1.07) Control Group: 12 (12/0) (11.66 ± 1.07)	Group 1: Functional Corrective Exercises Group 2: Corrective Plays	No Exercise	(8), (3), (NA)	Forward Head, Forward Shoulder, Kyphosis	Significant differences were observed in forward head, forward shoulder, and kyphosis in the intervention groups. Corrective plays had a higher positive effect on the forward head and kyphosis.
Maarouf et al., 2020 [13]	Basketball Players in Wheelchairs	Intervention group: 12 (12/0) (5.08 ± 39.80) Control Group: 12 (12/0) (11.23 ± 43)	Scapular Stability exercises	No Exercise	(8), (5), (60)	Forward Head, Forward Shoulder, Kyphosis	Significant improvement in forward head, forward shoulder, and kyphosis angles in the intervention group
Karimian et al., 2019 [23]	Teachers	Group: 12 (45.2 ± 8.1) Control Group: 11 (44.1 ± 7.8)	Corrective Exercises with Ergonomic Interventions	No Exercise	(12), (3), (45–60)	Forward Head, Forward Shoulder, Kyphosis	In exercise groups, significant differences were observed in the forward head, forward shoulder, and kyphosis angles.
Piri et al., 2021 [18]	Beautician women	Experimental Group: 20 (0/20) (33.40 ± 2.30) Control Group: 20 (0/20) (31.95 ± 1.47)	Myofascial Release, Stretching Exercises, and Dynamic Exercises	No Exercise	(12), (3), (60)	Forward Head, Forward Shoulder, Kyphosis	Significantly reduced forward head, forward shoulder, and kyphosis angles in the intervention group
Abdolahzade et al., 2019 [10]	Female Students	Intervention Group: 15 (0/15) (20.53 ± 1.55) Control Group: 15 (0/15) (20.00 ± 2.00)	Corrective Exercises based on NASM	No Exercise	(8), (3), (30–70)	Forward Head, Forward Shoulder, Kyphosis	Significant improvement was found in the NASM group’s forward head, forward shoulder, and kyphosis angles.
Hajizadeh et al., 2021 [11]	Martial Artists	Intervention group: 15 (15/0) (24.20 ± 4.12) Control Group: 15 (15/0) (24.66 ± 3.56)	General corrective Exercises	No Exercise	(10), (3), (30–70)	Forward Head, Forward Shoulder, Kyphosis	Significant changes in forward head, forward shoulder, and kyphosis angles in the experimental group
Cheshomi et al., 2017 [24]	Female volleyball and handball players	Intervention group: 18 (18/0) (29.18 ± 4.92) Control Group: 18 (18/0) (27.11 ± 5.74)	Strengthening and stretching exercises	No Exercise	(6), (3), (NA)	Forward Head, Forward Shoulder, Kyphosis	Significant changes in forward head, forward shoulder, and kyphosis angles in the experimental group
Hosseini and et al., 2016 [25]	Male Students	General corrective exercise group: 15 (15/0) (23.07 ± 2.03) Vibration group: 15 (15/0) (23.05 ± 2.03) Control Group: 15 (15/0) (24.40 ± 2.10)	Group 1: General corrective exercises Group 2: corrective exercises combined with vibration	No Exercise	(6), (3), (30–60)	Forward Head, Forward Shoulder, Kyphosis	Significant reduction in forward head, forward shoulder, and kyphosis angles was observed in both intervention groups—the vibration group showed greater improvement .
Haji Hosseini et al., 2014 [12]	Female Students	Strengthening exercise group: 10 (10/0) (21.50 ± 1.08) Stretching exercise group: 10 (10/0) (22.10 ± 1.80) Combined exercise group: 10 (10/0) (22.08 ± 1.97) Control Group: 10 (10/0) (22.90 ± 2.07)	Group 1: Strength Exercises Group 2: Stretching Exercises Group 3: Combined Exercises	No Exercise	(6), (3), (30–70)	Forward Head, Forward Shoulder, Kyphosis	Significant reduction in forward head, forward shoulder, and kyphosis angles was observed in all exercise groups, with the combination group showing the most significant reduction.
Miri et al., 2022 [26]	Female Students	Corrective exercise group: 8 (8/0) (16.75 ± 0.07) Postural education group: 8 (8/0) (15.50 ± 0.75) Combined group: 8 (8/0) (16.75 ± 1.03) Control Group: 8 (8/0) (16.00 ± 1.19)	Group 1: corrective exercises Group 2: postural training Group 3: corrective exercises and postural training	No Exercise	(8), (3), (60)	Forward Head, Forward Shoulder, Kyphosis	Significant differences were observed in forward head, forward shoulder, and kyphosis angles in all intervention groups.
Javazi et al., 2019 [27]	Female Students	Intervention group: 12 (12/0) (22.00 ± 1.53) Control Group: 12 (12/0) (21.58 ± 1.88)	Corrective exercises with physioball	No Exercise	(6), (3), (60)	Forward Head, Forward Shoulder, Kyphosis	Significant differences were observed in the intervention group’s forward head, forward shoulder, and kyphosis angles.
Ahmadi et al, 2022 [28]	Female Students	Intervention Group: 12 (0/12)( 22.4 ± 1.3) Control Group: 12 (0/12)( 22.2 ± 0.33)	Aquatic Corrective Exercises	No Exercise	8), (3), (50–70)	Forward Head, Forward Shoulder, Kyphosis	Significantly reduced forward head, forward shoulder, and kyphosis angles in the aquatic exercise group
Sarvari et al., 2022 [29]	Students	Intervention group: 20 (0/20)( 14.54 ± 1.82 ) Control group: 20 (0/20)( 14.43 ± 1.16)	Online Corrective Exercises	No Exercise	(8), (3), (30–45)	Forward Head, Kyphosis	Significant improvements in forward head and kyphosis angles in intervention group
Seidi et al., 2020 [17]	Students	Intervention group: 12 (12/0)( 25.3 ± 2.5 ) Control group: 12 (12/0)( 25.4 ± 1.5)	Comprehensive Corrective Exercises	No Exercise	(8), (3), (NA)	Forward Head, Forward Shoulder, Kyphosis	No exercise, comprehensive corrective exercises
Yaghoubitajani et al., 2022 [30]	office workers	Workplace group: 12 ( 37.00 ± 8.12) Online-supervised group: 12 ( 38.58 ± 7.34) Control group: 12 ( 38.91 ± 3.87)	Group 1: corrective exercises at the workplace Group 2: Online-supervised : corrective exercises	No Exercise	(8), (3), (50–60)	Forward Head, Forward Shoulder, Kyphosis	both intervention groups improved from baseline to follow-up for Forward Head, Forward Shoulder, and Kyphosis.
Guo et al., 2023 [31]	College students	Intervention group: 20 (11/9)( 19.00 ± 0.97) Control group: 20(7/13)( 18.85 ± 0.88)	cervical and thoracic “Daoyin” training.	No Exercise	(8), (5), (NA)	Forward Head, Forward Shoulder	obvious improvement in the forward head angle, forward shoulder angle,
Park et al., 2014 [32]	children	Intervention group: 20 (10/10)( 13.55 ± 2.21) Control group: 20(10/10)( 13.75 ± 1.80	Strengthening and stretching exercise	No Exercise	(25), (3), (NA)	Forward Head, Forward Shoulder	The complex training improved posture
Firouzjah et al., 2023 [33]	volleyball players	Intervention group: 15 ( 16/46 ± 0/63) Control group: 15 ( 16/80 ± 0/77)	Strengthening and stretching exercise	No Exercise	(10), (3), (30–70)	Forward Head, Forward Shoulder, Kyphosis	significant good effect on forward head, forward shoulder, and kyphosis.
Ruivo et al., 2016 [34]	adolescents	Intervention group: 42(16/26) (15.5 ± 1.0) Control group: 46(15/31) (15.9 ± 1.1)	Strengthening and stretching exercise in addition to Physical Education classes	Physical Education classes	(32), (2), (NA)	Forward Head, Forward Shoulder	The exercise intervention successfully decreased the forward head and protracted shoulder in adolescents.
Lynch et al.,2010 [5]	National Collegiate Athletic	Intervention group: 14 (19.29 ± 1.44) Control group: 14 (19.29 ± 1.20)	Strengthening and stretching exercise	No Exercise	(8), (3), (NA)	Forward Head, Forward Shoulder	The exercise intervention was successful at decreasing forward head and rounded shoulder
Nitayarak et al., 2021 [35]	women	Intervention group: 19(0/20) (20.26 ± 1.20) Control group: 20(0/20) (20.15 ± 1.27)	d scapular stabilization exercises using elastic bands	No Exercise	(4), (3), (NA)	Forward Head, Forward Shoulder, Kyphosis	significant improvement in the cervical and shoulder angle
Shalamzari et al., 2022 [36]	formal nurses	Intervention group: 31(31/0) (34.77 ± 9.19) Control group: 31(31/0) (37.12 ± 8.76)	Strengthening and stretching exercise	No Exercise	(8), (3), (30–70)	Forward Head, Kyphosis	Significant improvements in forward head and kyphosis angles in the intervention group

### Quality assessment

We utilized the JBI checklist to assess the quality of the studies. As this checklist provides separate criteria for different study types, we employed the quasi-experimental and clinical trial checklists for the studies included in this article. The assessment results are presented in a detailed Table 2.

**Table 2:** Score of Quality Assessment

study	Q1	Q2	Q3	Q4	Q5	Q6	Q7	Q8	Q9	Q10	Q11	Q12	Q13	Total
**Semi Experimental Studies**														
Hajihosseini et al., 2015 [12]	1	1	1	1	1	0	1	1	--	--	--	--	--	7
Cheshomi et al., 2018 [24]	1	1	1	1	1	0	1	1	--	--	--	--	--	7
Karimian et al., 2019 [23]	1	1	1	1	1	0	1	1	--	--	--	--	--	7
Abdolahzade et al., 2019 [10]	1	1	1	1	1	0	1	1	--	--	--	--	--	7
Hajihoseini et al., 2017 [25]	1	1	1	1	1	0	1	1	--	--	--	--	--	7
Javazi et al., 2019 [27]	1	1	1	1	1	0	1	1	--	--	--	--	--	7
Maarouf et al., 2020 [13]	1	1	1	1	1	0	1	1	--	--	--	--	--	7
Salamat et al., 2020 [22]	1	1	1	1	1	0	1	1	--	--	--	--	--	7
Hajizadeh et al., 2021 [11]	1	1	1	1	1	0	1	1	--	--	--	--	--	7
Ahmadi et al., 2022 [37]	1	1	1	1	1	0	1	1	--	--	--	--	--	7
Sarvari et al., 2022 [29]	1	1	1	1	1	1	1	1	--	--	--	--	--	8
Miri et al., 2022 [26]	1	1	1	1	1	0	1	1	--	--	--	--	--	7
Park et al.,2014 [32]	1	1	1	1	1	0	1	1	--	--	--	--		7
Firouzjah et al., 2023 [33]	1	1	1	1	1	0	1	1	--	--	--	--	--	7
Shalamzari et al., 2022 [36]	1	1	1	1	1	0	1	1	--	--	--	--	--	7
**Randomized Controlled Trials**														
Seidi et al., 2020 [17]	1	0	1	1	0	1	0	1	1	0	1	1	1	9
Piri et al., 2021 [18]	1	0	1	1	0	1	1	1	1	0	1	1	1	10
Nitayarak et al., 2021 [35]	1	0	1	1	0	1	1	1	1	0	1	1	1	10
Lynch et al.,2010 [5]	1	0	1	1	0	1	0	1	0	0	1	1	1	8
Ruivo et al., 2016 [34]	1	0	1	1	0	1	1	1	1	1	1	1	1	11
Guo et al., 2023 [31]	1	0	1	1	0	1	1	1	1	0	1	1	1	10
Yaghoubitajani et al., 2022 [30]	1	0	1	1	0	1	0	1	1	0	1	1	1	9

### Statistical analyses

In the current study, we employed the Comprehensive Meta-Analyses (CMA ver3) statistical software for data analysis. We gathered data from the articles selected for the study, including pre-and post-test means, standard deviation, *P*-values, sample sizes, and mean differences (if obtainable). Data heterogeneity was assessed using the I^2^ and Q test. Funnel Plot and Egger test were utilized to evaluate publication bias. In instances where significant publication bias was detected, we utilized the trim-and-fill method to gauge the possible effect on the final meta-analysis results and to determine the extent of studies missed in this domain.

## Results

**Fig. 1:**
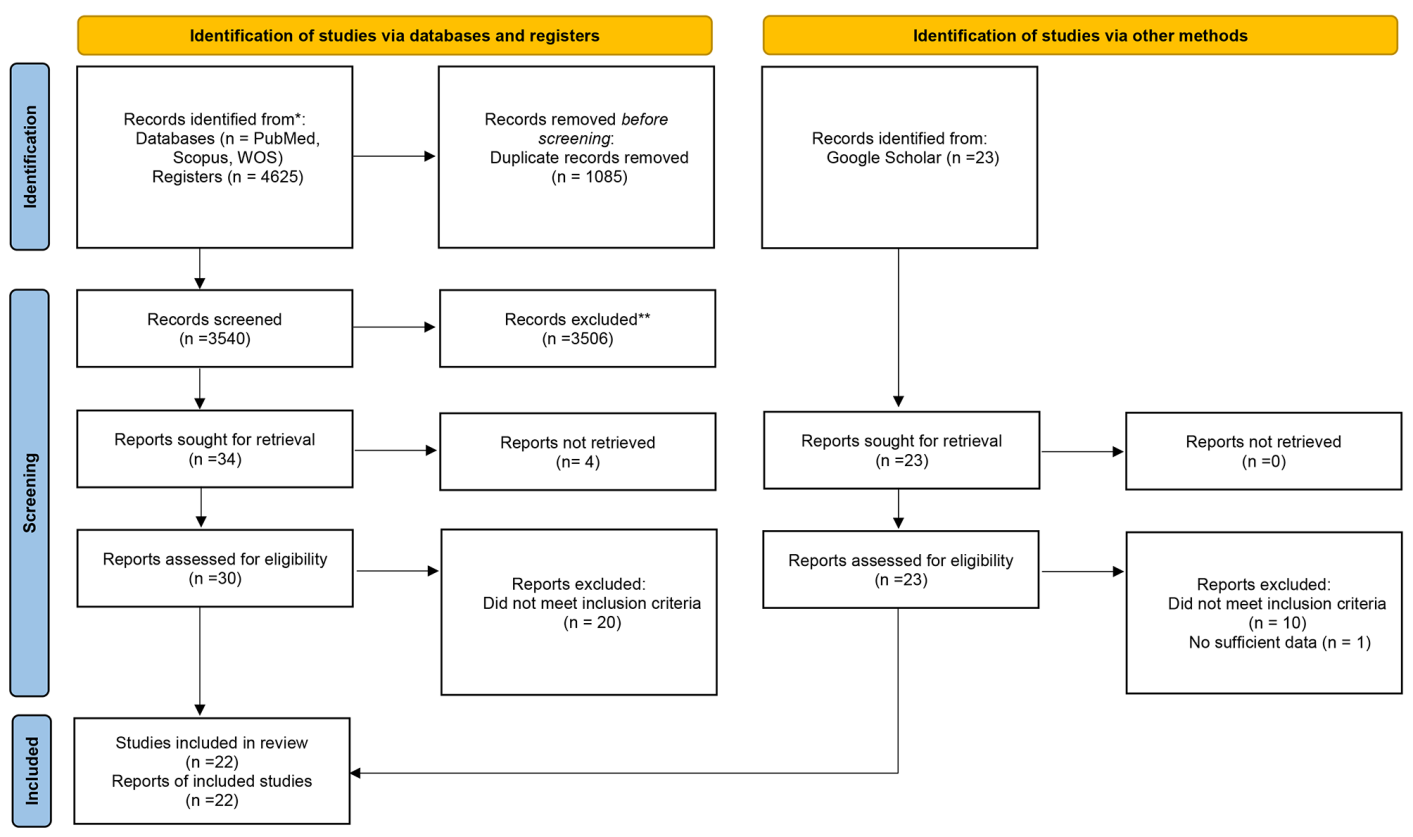
Search and selection of studies for systematic review according to PRISMA guidelines

There were a total of 4625 articles identified in the selected databases. Once the data was entered into EndNote software and duplicate records were removed, 3540 articles remained. After reviewing the abstracts and titles, 34 articles were selected for further analysis, and the remaining articles were excluded. Following this, the complete text of the 30 chosen articles was carefully analyzed; ultimately, ten papers were deemed suitable for the study. Additionally, 12 studies from the Google Scholar database were included, resulting in 22 studies. Each of the three abnormalities associated with UCS (forward head posture, rounded shoulders, and hyper-kyphosis) were analyzed separately to aid in data analysis. The effect of therapeutic exercise has been the subject of research about UCS. In this study, in the 22 eligible studies, a total of 903 participants were involved, and this data is presented in Fig. 1.

### Forward head posture

**Fig. 2:**
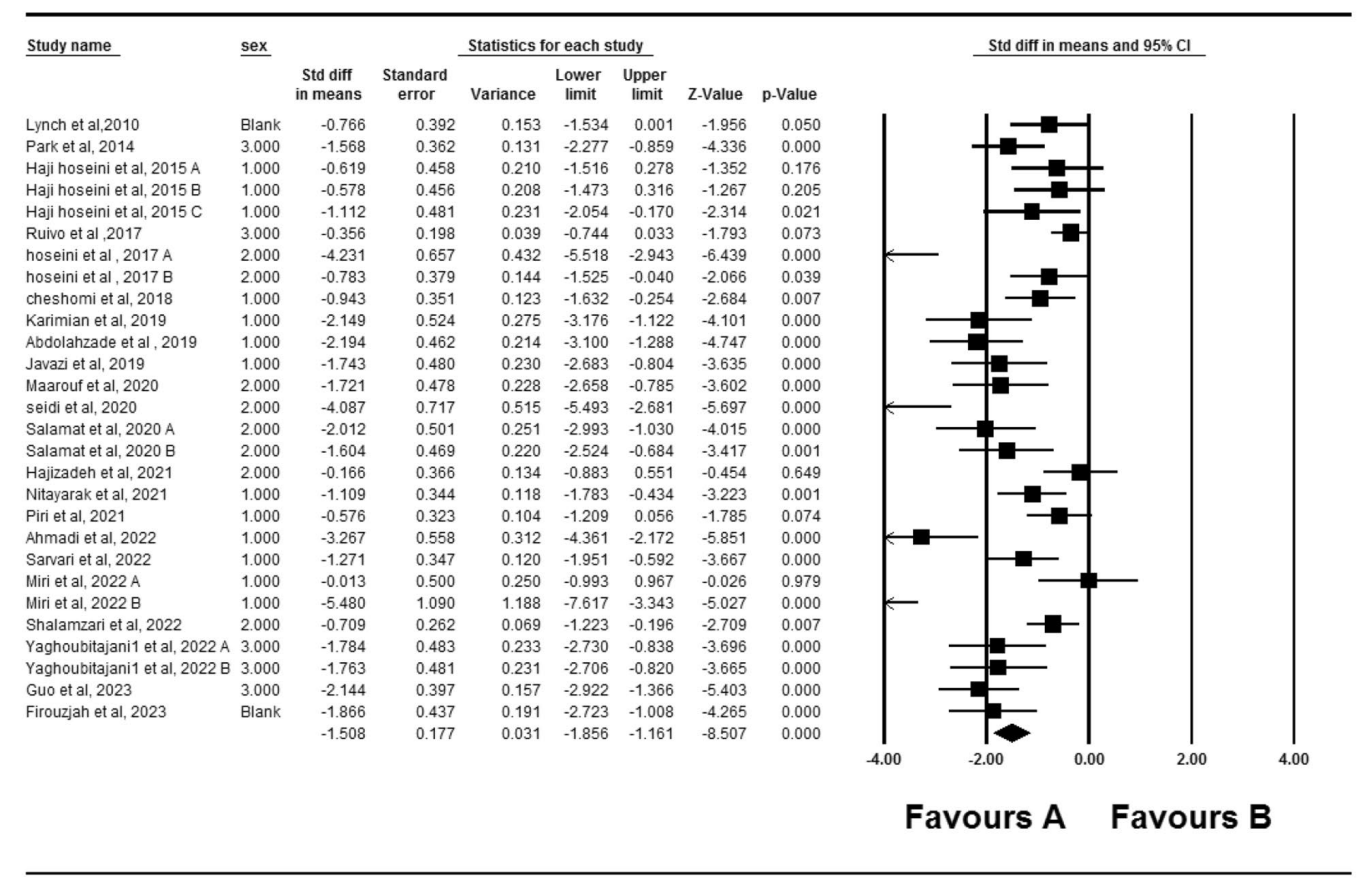
Forest plot of the effect of therapeutic exercise on Forward head posture

**Fig. 3:**
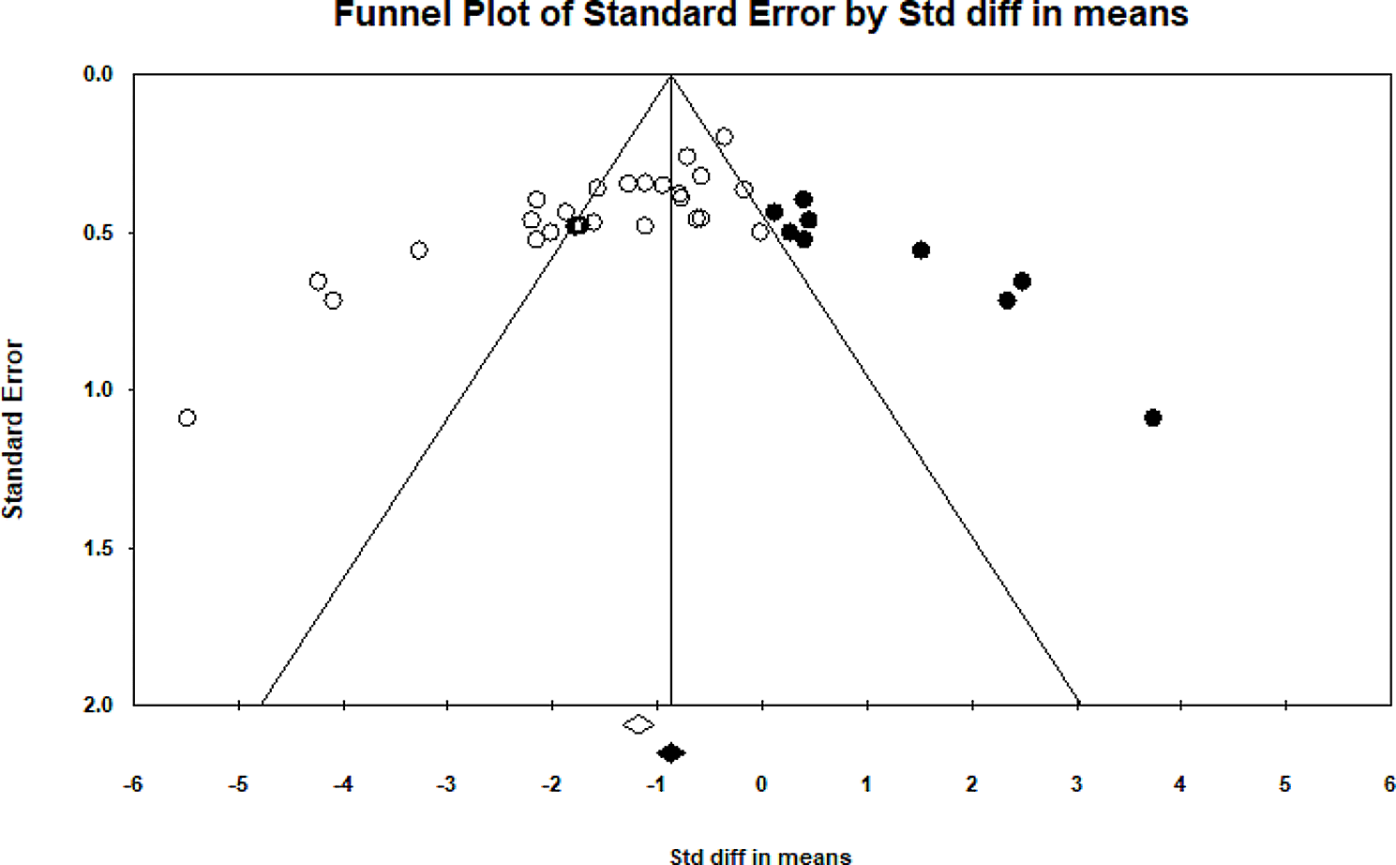
Funnel plot of studies worked on forward head posture

Twenty- two eligible studies have investigated the impact of therapeutic exercise on forward head posture [5, 10–13, 17, 18, 22–36]. Of these studies, three studies had two independent intervention groups that were analyzed. In total, 903 participants took part in these studies. Forest plot analysis of the data showed a significant improvement in forward head posture with therapeutic exercise (CI 95% = -1.85–1.161, *P* = 0.001) (Fig. 2). The I2 and Q tests were used to assess heterogeneity, which showed significant heterogeneity (*P* = 0.001) (I2 = 80.39) among data. Funnel plot and Egger’s tests suggested the possibility of publication bias (*P* = 0.001) (Fig. 3). Furthermore, the results of the Trim and Filled also indicated that adding nine random articles cannot change the overall results of this study. Because of the heterogeneity, meta-regression was used to examine the possible effect of age on the eligible studies’ results. Age and weight did not significantly impact the studies’ results (*P* < 0.05).

### Rounded shoulders

**Fig. 4:**
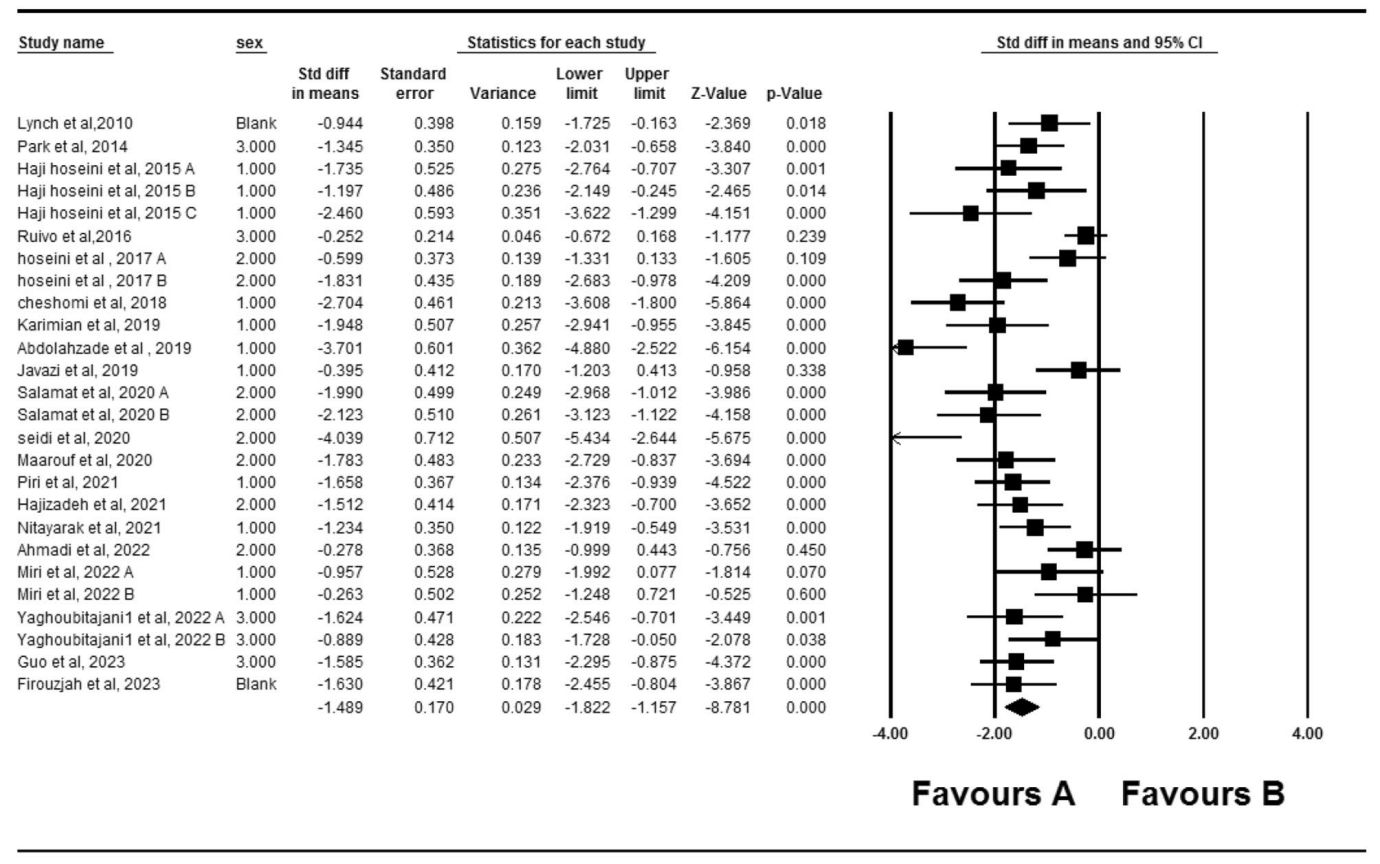
Forest plot of the effect of therapeutic exercise on Rounded shoulders

**Fig. 5:**
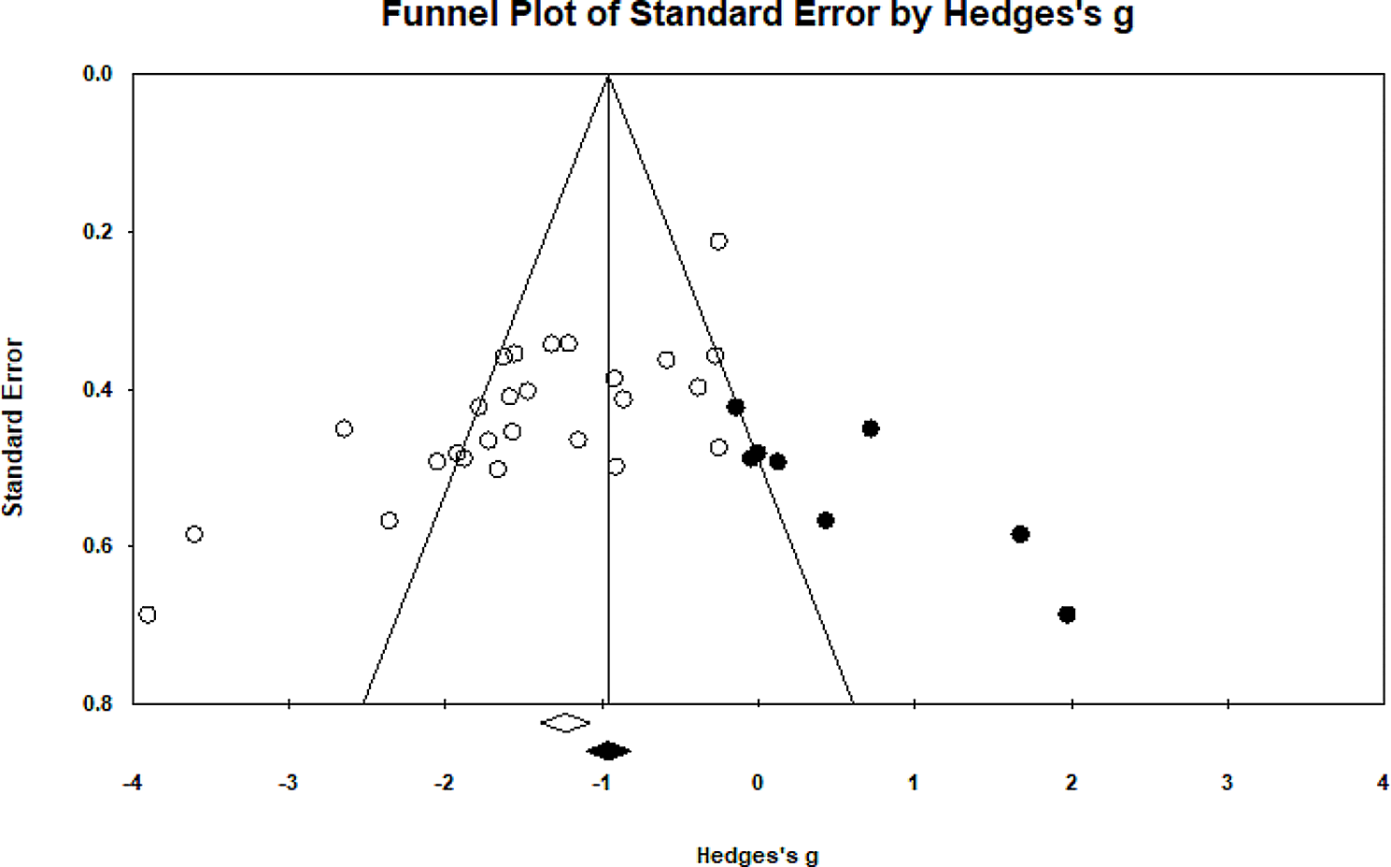
Funnel plot of studies worked on Rounded shoulders

Twenty studies have examined the impact of therapeutic exercise on rounded shoulders [5, 10–13, 17, 18, 22–28, 30–35]. Two of these studies had two independent intervention groups, while one study had three independent intervention groups. A total of 774 participants were involved in these studies. Forest plot analysis of the data showed a significant improvement in rounded shoulders with therapeutic exercise (*P* = 0.001, CI 95%=-1.822–1.157) (Fig. 4). After assessing heterogeneity, significant heterogeneity was observed (*P* = 0.001) (I2 = 75.38). Funnel plot and Egger’s tests indicated publication bias in the studies (*P* = 0.001) (Fig. 5). Furthermore, the results of the Trim and Filled also showed that adding eight random articles cannot change the overall results of this study. Meta-regression was used to examine the possible effect of age on the eligible studies’ results. Age and weight did not significantly impact the studies’ results (*P* < 0.05).

### Thoracic kyphosis

**Fig. 6:**
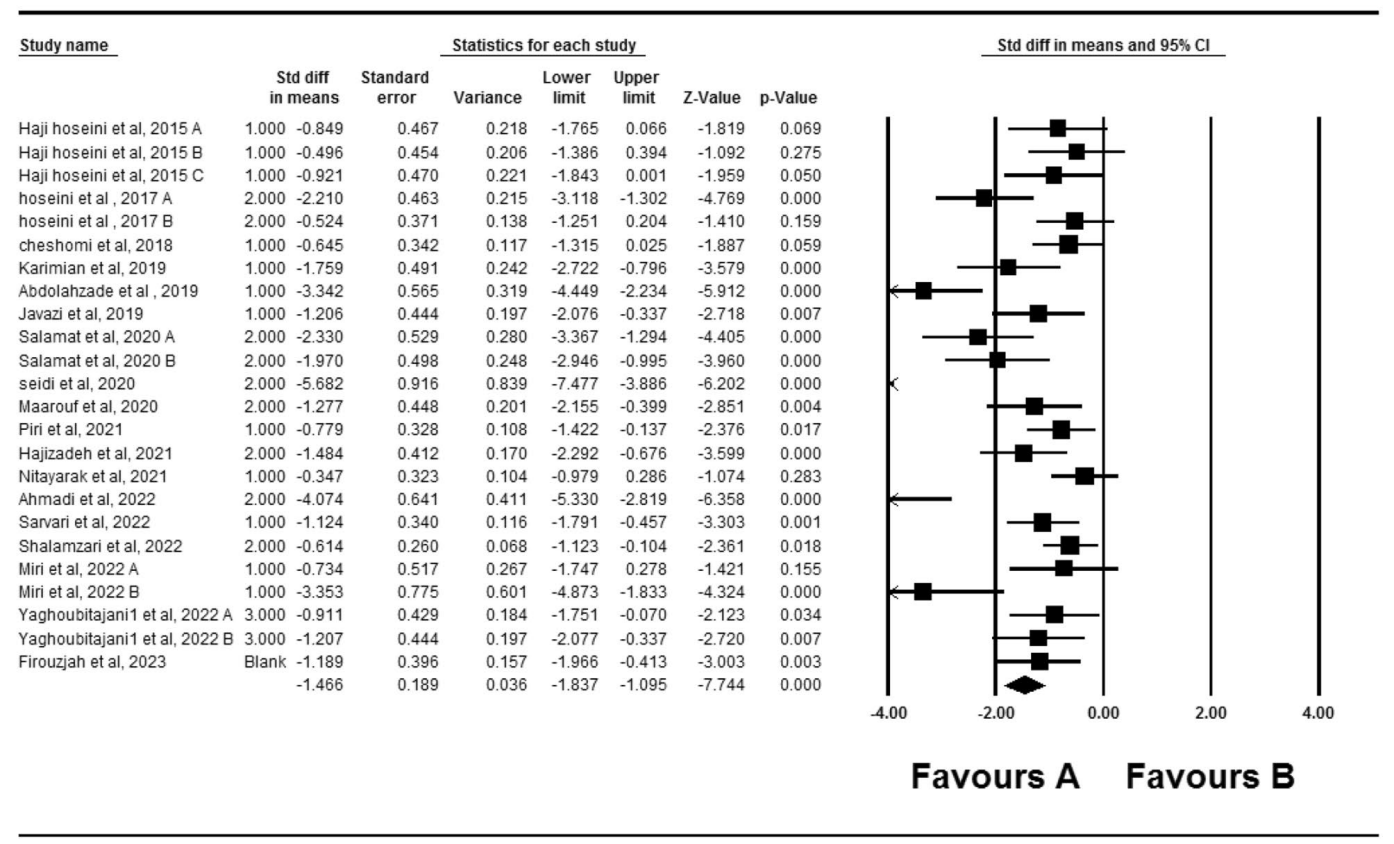
Forest plot of the effect of therapeutic exercise on Thoracic Kyphosis

**Fig. 7:**
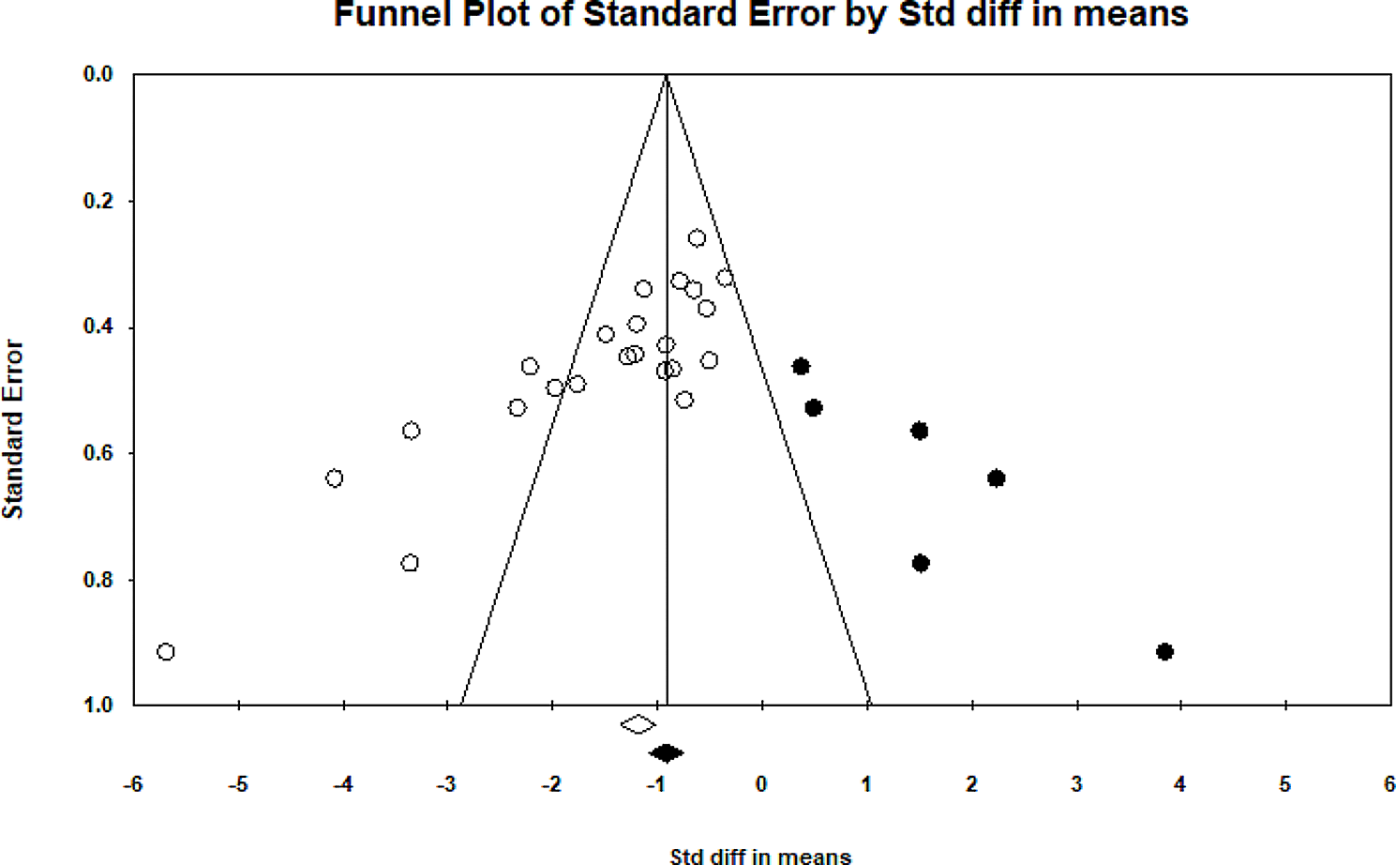
Funnel plot of studies worked on Thoracic kyphosis

Eighteen studies have examined the effect of therapeutic exercise on thoracic kyphosis [10–13, 17, 18, 22–30, 33, 35, 36]. In three studies, both independent intervention groups were utilized, while in one study, all three independent intervention groups were included. A total of 673 participants were involved in these studies. Data analysis using CMA and Forest plot analysis demonstrated a significant improvement in thoracic kyphosis with therapeutic exercise (*P* = 0.001, CI 95%= -1.83–1.09) (Fig. 6). After assessing heterogeneity, significant heterogeneity was observed (*P* = 0.001) (I2 = 77.86). Funnel plot and Egger’s tests indicated publication bias in the studies (*P* = 0.001) (Fig. 7). Furthermore, the results of the Trim and Filled also showed that adding six random articles cannot change the overall results of this study. Meta-regression was used to examine the possible effect of age and weight on the eligible studies’ results. It is demonstrated that age and weight did not significantly impact the studies’ results (*P* < 0.05).

## Discussion

The results of this study indicate that prescribing therapeutic exercises can be an effective intervention in modifying the angles of the forward head, rounded shoulder, and thoracic kyphosis. The meta-analysis results showed a significant difference in the change in the forward head angle between the groups participating in therapeutic exercise programs and the control groups.

The result of this study is consistent with other research indicating the effectiveness of therapeutic exercises in improving forward head posture in individuals with UCS. For instance, previous studies have demonstrated that therapeutic exercises using vibration devices [25], NASM-based corrective exercises [10, 23], strength, stretching, and a combination of both exercises, as well as comprehensive corrective exercises [17] all have a significant impact on forward head angle reduction in individuals with UCS. It seems that the better forward head posture may result from decreases in the anterior displacement of the head secondary to better mechanical advantage and biomechanical function of the neck muscles that contribute to cervical flexion [38, 39]. In this line, to address forward head posture, selected therapeutic exercises aimed to stretch the shortened muscles of the neck, such as the sternocleidomastoid, levator scapulae, scalenes, and pectoralis major, and to strengthen the deep neck flexor muscles, such as the longus colli, longus capitis, and anterior scalene. Additionally, exercises aimed to strengthen the thoracic spine and shoulder muscles to reduce the forward head angle [18, 19].

On the other hand, this study’s results indicate that therapeutic exercise significantly improves rounded shoulder posture in individuals with UCS. Previous studies have also demonstrated that various therapeutic programs, including two functional therapeutic programs and therapeutic games [22], NASM-based corrective exercises, selected corrective exercises [24], and strength, stretching, or a combination of both exercises [17], have a significant impact on improving rounded shoulder posture in individuals with UCS.

Ultimately, this study demonstrates that therapeutic exercise can significantly reduce the thoracic kyphosis angle in individuals with UCS. In support of this, various studies have shown that different therapeutic exercises can effectively mitigate the thoracic kyphosis angle in individuals with UCS [17, 18, 22, 24].

To explain why therapeutic exercises are effective in improving postural alignment in people with UCS, several points can be considered [2]. Muscle imbalances resulting from prolonged positioning or repetitive movements in a specific and static position can affect upper body posture. Biomechanical studies have shown that the deep flexor muscles of the neck, upper back extensors, and shoulder adductors become weakened and strained compared to their antagonists [40]. This leads to an imbalance in the upper quarter of the body, resulting in forward head posture, abnormal scapular protraction, increased thoracic kyphosis, and neck flexion, which can eventually lead to painful shoulders and shortening of anterior shoulder muscles, such as the pectoralis major and minor [35, 41]. These abnormalities are interrelated in a kinetic chain, and a significant change in the natural pattern of one of the spinal column curves can lead to compensatory changes in other curves [40]. Therefore, researchers have selected various therapeutic exercises that focus on the body’s chain reaction and simultaneously address the three abnormalities associated with UCS: forward head posture, rounded shoulders, and hyper-kyphosis. This seems to be the primary reason for the positive results found in the studies included in this review [17]. The rhomboid, trapezius, and serratus anterior muscles are the most important stabilizers of the shoulder, primarily controlling shoulder movements in a coordinated fashion [42]. In UCS, these muscles tend to weaken, and inhibition of the rhomboid and serratus anterior muscles can lead to increased shoulder abduction with anteriorly rotated shoulders [43]. According to Kendall [7], this abnormality can result from the shortening of the pectoralis minor muscle and weakness of the middle trapezius muscle. Additionally, in anterior shoulder dysfunction, the humerus bone is influenced by its connection to the glenoid cavity, causing the arm to move forward and rotate internally. This condition can result in weakness and strain of the shoulder’s external rotator muscles and shortening of the internal rotator muscles of the shoulder [7]. Therefore, exercise interventions in the reviewed studies may have helped reduce UCS by creating balance in muscular activity.

Moreover, this analysis revealed significant heterogeneity in the findings of different studies across all three variables of forward head posture, rounded shoulder, and thoracic kyphosis. It is worth noting that these studies were conducted on individuals with various age ranges, jobs, and interventions employed to improve UCS. Therefore, these factors may contribute to the heterogeneity in the results of the studies included in the analysis. In this context, the meta-regression results demonstrated that age and weight did not significantly affect the studies’ results.

When generalizing the findings of this study, it is essential to consider some of its limitations. Firstly, most studies have not focused on investigating the long-term effects of therapeutic exercise on UCS, so it is unclear how long-lasting the effects mentioned in this article would be. Secondly, this study aimed to investigate the effects of therapeutic exercises on the postural alignment of individuals with UCS. It did not examine the impact of therapeutic exercises on performance, psychological, or social characteristics. Thirdly, it should be noted that the UCS is a cluster of symptoms, including postural deviations, specific patterns of muscle tightness and weakness, and scapular dyskinesia. However, it should be considered that most articles have relied solely on postural variables for diagnosing UCS. Therefore, they may lack the necessary accuracy in identifying this condition. Moreover, in this study the quality assessment was not limited to JBI score cut-off value and had no limitations regarding the mean age or the participants’ jobs. This point may have resulted in heterogeneity among the meta-analysis results, and therefore, caution should be exercised in generalizing these findings to other groups. Ultimately, this study only assessed Persian and English-language peer-reviewed journals and did not include other scientific literature like conference proceedings, books, and textbook chapters. It may be worthwhile to consider a broader range of academic sources and include materials in different languages to broaden the scope of future research.

## Conclusion

The results revealed that prescribing various therapeutic exercises effectively improves postural alignment in individuals with UCS. Exercise prescription can significantly improve such individuals’ forward head posture, rounded shoulder, and thoracic kyphosis angles. Moreover, it seems that the effectiveness of therapeutic exercise for this condition may be slightly less in children than in adults.

### Electronic supplementary material

Below is the link to the electronic supplementary material.

**Supplementary Material 1:** The datasets generated and analysed during the current study

**Supplementary Material 2:** Raw data reviewd in this paper from various database

## Data Availability

The datasets generated and analyzed during the current study are available in supplementary file 1, and the search results from various databases are presented in supplementary file 2.
